# Supplementary figures and images for: Mapping of a Major-Effect Quantitative Trait Locus for Seed Dormancy in Wheat
Source: Int J Mol Sci. 2024 Mar 26;25(7):3681. doi: 10.3390/ijms25073681 (PMC11011268; doi:10.3390/ijms25073681)

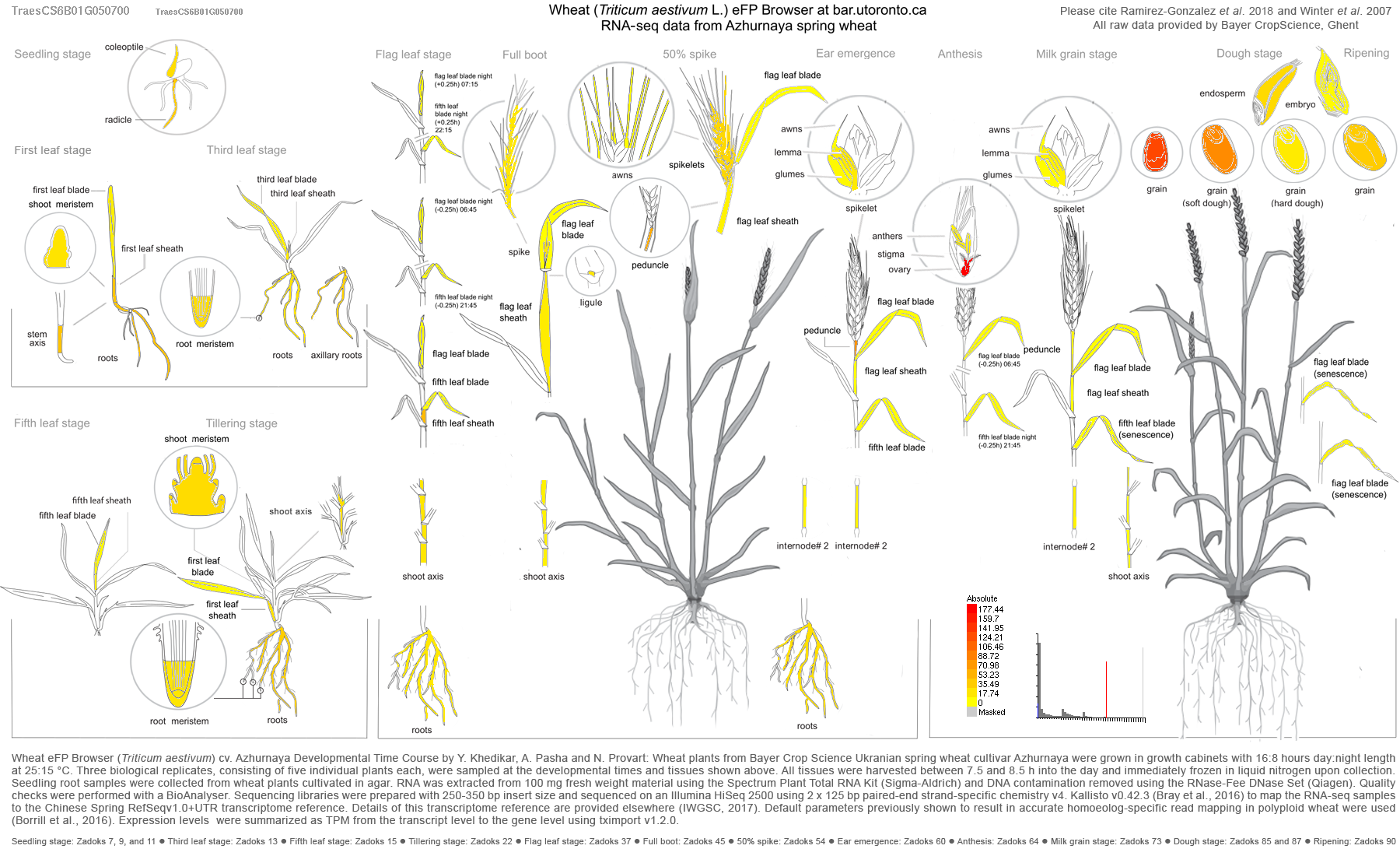

Supplement: Supplementary file 1 [file ijms-25-03681-s001.zip › FigureS1.png]
